# Parameter calibration method for car-following models under snowy weather conditions: Integrating an informer time series encoder and physics-informed neural networks

**DOI:** 10.1371/journal.pone.0350550

**Published:** 2026-06-09

**Authors:** Yaping Sun, Wenfang Li, Mengyang Yang, Xingchen Zhang

**Affiliations:** 1 School of Intelligent Transportation and Intelligent Construction Engineering, Huanghe Jiaotong University, Jiaozuo, China; 2 School of Traffic and Transportation, Beijing Jiaotong University, Beijing, China; National University of Singapore, SINGAPORE

## Abstract

Under snowy weather conditions, factors such as road conditions and weather conditions significantly affect vehicle car-following behavior. Traditional car-following models struggle to accurately capture driving characteristics on slippery roads. To address this, this paper proposes a parameter calibration method for car-following models under snowy conditions, considering factors including road adhesion coefficient and visibility. Five classical car-following models are selected for analysis: the GM model, the Gipps model, the Intelligent Driver Model (IDM), the Wiedemann model, and the Full Velocity Difference Model (FVDM). A systematic analysis is conducted on the key parameters to be calibrated for each model in snowy environments. To overcome the poor adaptability and low accuracy of traditional calibration methods, an adaptive parameter calibration framework combining an Informer time series encoder and a physics-informed neural network is proposed. This method extracts features of snowy environments using the Informer time series encoder and achieves dynamic optimization of model parameters via the physics-informed neural network algorithm, making it applicable to multiple car-following models simultaneously. Validation results based on the NGSIM dataset and real vehicle test data under snowy conditions show that the proposed method improves calibration accuracy by 12.7% under snowy scenarios compared to the traditional genetic algorithm, and exhibits strong generalization capability across different car-following models. This research can provide fundamental models for traffic simulation systems and enhance simulation accuracy.

## 1. Introduction

Snowy weather is one of the major meteorological factors affecting highway traffic safety and operational efficiency. According to statistics, traffic accidents caused by snowy conditions account for more than 25% of all crashes on highways in northern China, among which rear‑end collisions constitute about 60% [1]. Car‑following behavior is a core component of microscopic traffic flow simulation; its accuracy directly determines the capability of traffic flow models to represent traffic operation under snowy environments. However, under such conditions, the road adhesion coefficient decreases significantly, visibility drops, driver perception‑reaction time increases, following distances enlarge, and acceleration/deceleration behavior becomes more conservative, rendering model parameters calibrated under dry pavement conditions invalid. Therefore, developing precise calibration methods for car‑following model parameters under snowy weather is of great significance for improving traffic simulation accuracy and optimizing dynamic traffic management strategies.

Parameter calibration of car‑following models serves as a crucial bridge linking theoretical models and real‑world driving behavior [2]. Its accuracy directly affects the reliability of traffic flow simulation, the effectiveness of vehicle dynamics control, and the trustworthiness of virtual validation for autonomous driving systems [3]. Parameter calibration combines optimization theory and statistical inference: by constructing an appropriate objective function, selecting a suitable optimization algorithm, and using high‑precision trajectory data, it inverts the parameter combination that best matches the model output to the measured data.

However, as research deepens, the academic community has gradually recognized the complexities hidden in this seemingly standardized procedure [4]. Traditional calibration studies have mostly focused on deterministic models, such as the Intelligent Driver Model (IDM), the Optimal Velocity Model (OVM), or the Gipps model. These models assume that the same input always yields the same output, so calibration efforts mainly concentrate on how to choose the objective function, handle data noise, and improve optimization efficiency [5–8]. Regarding the choice of objective function, researchers have found that using spacing error as the core metric is often more robust than using speed error, because spacing, as an accumulated variable, can smooth out high‑frequency noise [9]. At the optimization algorithm level, methods such as genetic algorithms, sequential quadratic programming, least squares, maximum likelihood estimation, and particle swarm optimization each have their applicable scenarios, and algorithm performance strongly depends on the specific data characteristics and model structure [10]. Li et al. [11] adopted a genetic algorithm to calibrate the IDM parameters, achieving good results under dry pavement. Treiber and Kesting proposed a Bayesian inference‑based calibration method that can quantify parameter uncertainty [7]. In recent years, deep learning approaches have gradually been applied to traffic modeling; for instance, Mo et al. [12] used LSTM networks to directly learn car‑following behavior, bypassing the constraints of traditional model structures. Moreover, data sampling intervals, trajectory completeness, interaction effects among parameters, and the global sensitivity of the model itself all systematically influence calibration results, prompting researchers to introduce global sensitivity analysis to identify key parameters, thereby enabling model simplification or hierarchical calibration [13].

In recent years, with a growing understanding of the stochastic nature of driving behavior, stochastic car‑following models have become a research hotspot [14–16]. Unlike deterministic models, stochastic models explicitly introduce random terms into the dynamic equations to capture the inherent variability in drivers’ perception, decision‑making, and execution processes [14]. For example, the Laval model describes the distribution of desired acceleration using a truncated normal distribution [15], while the Treiber model adds a stochastic process to the IDM [7]. However, the introduction of stochasticity makes calibration exceedingly complex [17]. Wagner et al. [9] pointed out that even if the calibration procedure is theoretically flawless, parameter estimation may still suffer systematic failures because the randomness within the model couples with data noise, making it difficult for traditional error‑minimization methods to distinguish parameter bias from stochastic fluctuations. To address this issue, Zhou et al. [1] recently proposed a method called “Minimum Error over Multiple Runs” (MRMin). Its core idea is to perform multiple stochastic simulations, find the parameter set that minimizes the error in each run, and then take statistical characteristics of the multiple results as the final estimate. Compared with traditional methods like Mean Error over Multiple Runs (MRMean) and Maximum Likelihood Estimation (MLE), MRMin can effectively avoid the tendency to “degenerate” a stochastic model into a deterministic one, achieving nearly unbiased parameter estimation [18–20]. More importantly, combined with Bootstrap sampling, this method can estimate the covariance matrix of the parameters, thereby providing a foundation for confidence interval construction and statistical inference [21]. This marks a shift from pure parameter optimization to a systematic framework that includes uncertainty quantification.

From the current state of research, the field of car‑following model parameter calibration is undergoing profound transformations: from deterministic to stochastic, from point estimation to probabilistic inference, and from single‑model to hybrid modeling. In stochastic model calibration, methods like MRMin have preliminarily solved the problem of biased parameter estimation caused by model randomness, but their generality in complex urban road scenarios and multi‑vehicle interaction situations still needs validation [22]. Regarding uncertainty quantification, although researchers have begun to pay attention to estimating the parameter covariance matrix, the propagation mechanism of parameter uncertainty still lacks a systematic analytical framework [23]. In terms of data fusion, with the increasing accumulation of naturalistic driving datasets (e.g., HighD, exiD) and closed‑field test data, there remains a lack of unified theoretical guidance on how to rationally fuse data from different sources, with different accuracies and sampling characteristics, during the calibration process [24]. Furthermore, the integration of calibration and validation is becoming increasingly evident. Researchers have started to realize that simply maximizing the goodness of fit is insufficient to guarantee the model's validity in virtual test scenarios [25]. A closed‑loop feedback mechanism must be established, linking calibration data selection, objective function design, and validation scenario construction, so that the calibration process is deeply aligned with the ultimate application goals. Overall, car‑following model parameter calibration is no longer a pure optimization computation problem; it has evolved into a comprehensive engineering science problem integrating statistics, system identification, uncertainty quantification, and application‑scenario validation. Future research needs to continue making progress in algorithm robustness, uncertainty propagation mechanisms, and cross‑scenario generalization.

Parameter calibration methods mainly include least‑squares‑based error minimization, maximum likelihood estimation, and Bayesian inference. These methods have achieved good results for deterministic models, but their limitations become increasingly evident when facing scenarios with strong nonlinearity, high noise, and dynamically changing conditions, such as snowy weather. In recent years, some scholars have attempted to introduce data‑driven methods for parameter identification. For example, using neural networks to directly fit the mapping between model parameters and driving environments, or employing recursive least squares for online parameter updating [26–27]. To some extent, these studies have improved the ability to respond to environmental changes, but two problems remain. First, most methods are designed for a specific car‑following model; when the model structure changes, the calibration framework must be redesigned, lacking generality. Second, purely data‑driven methods struggle to embed physical prior knowledge, and under extreme snowy conditions where training data are insufficient, they may produce parameter estimates that violate physical laws [28]. Moreover, most existing studies treat “snowy weather” merely as a discrete operational label, distinguishing dry pavement from snowy pavement by setting different parameter sets, without fully exploiting the continuously changing dynamic characteristics of the snowy environment. Key environmental variables such as road adhesion coefficient, visibility, and snow depth change continuously in real driving scenarios, and their effects on car‑following behavior are nonlinear and hysteretic. Existing calibration methods lack explicit extraction and fusion mechanisms for these environmental features, so the calibration results cannot adaptively adjust as the environment changes [29].

To address the above shortcomings, this paper proposes a parameter calibration framework that integrates an Informer time series encoder with a Physics‑Informed Neural Network (PINN). The Informer time series encoder employs a ProbSparse self‑attention mechanism to extract long‑range temporal features of car‑following behavior, generating a compact driving behavior representation vector. The physics‑informed neural network feeds the temporal features into a parameter generation network, while simultaneously incorporating the differential equations of the car‑following model as physical residual constraints into the loss function, thereby obtaining physically plausible model parameters through joint optimization. This framework is model‑agnostic and can be simultaneously applied to various classical car‑following models such as IDM, Gipps, and OVM, simply by mapping the parameter space of the corresponding model to the action output of the reinforcement learning component. In this way, the proposed method overcomes the static limitations of traditional calibration methods under time‑varying environments, achieving a leap from “fixed parameters” to “environmentally adaptive parameters”, and provides a new technical path for high‑fidelity vehicle simulation and autonomous driving decision‑making under complex weather conditions such as ice and snow.

## 2. Vehicle following characteristics in snow weather

The impact of snowy environments on car‑following behavior is mainly reflected in three aspects: the driver's perception ability, decision‑making logic, and vehicle physical response characteristics. Compared with dry pavement, under snowy conditions the road adhesion coefficient decreases significantly, visibility drops, and braking distance increases considerably. These objective environmental changes directly translate into systematic alterations in car‑following behavior characteristics.

Under snowy weather, drivers generally adopt conservative driving strategies, actively reducing speed to gain greater safety margins. Extensive naturalistic driving data indicate that average speeds under snowy conditions are about 15% to 30% lower than on dry pavement, with speed distributions being more concentrated and higher‑speed driving behavior significantly reduced [5]. Measured data show that the average time headway under snowy conditions can increase from 1.5‑2.0 seconds on dry pavement to 2.5‑4.0 seconds [10]. Some simulator experiments have found that driver reaction time under snowy conditions can increase by 0.3 to 0.8 seconds [11]. In car‑following models, this change is reflected as an increase in the reaction time parameter, which has a particularly notable impact on models that explicitly include reaction delays. The friction coefficient *μ* on snowy roads can drop from 0.7‑0.8 on dry pavement to 0.1‑0.3, resulting in a significant extension of vehicle braking distance. According to the kinematic formula, the braking distance s at initial speed *v*₀ is *s* = *v*₀²/(2 *μg*); when *μ* decreases from 0.7 to 0.2, the braking distance increases to 3.5 times its original value. During snowfall, visibility typically drops to 100‑500 meters, affecting the driver's ability to accurately perceive speed changes of the leading vehicle and leading to prolonged reaction times. When visibility is below 200 m, driver reaction time can extend from the normal range of 1.0‑1.5 seconds to 2.0‑3.0 seconds [5].

**Fig 1:**
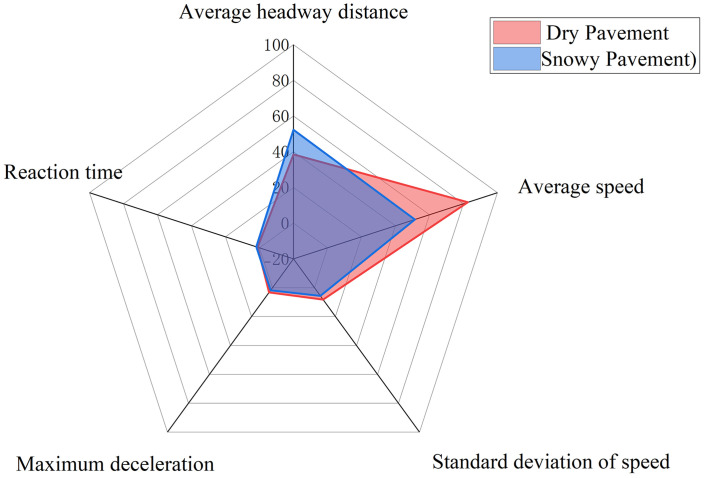
Traffic parameters under snowy weather.

**Fig 2:**
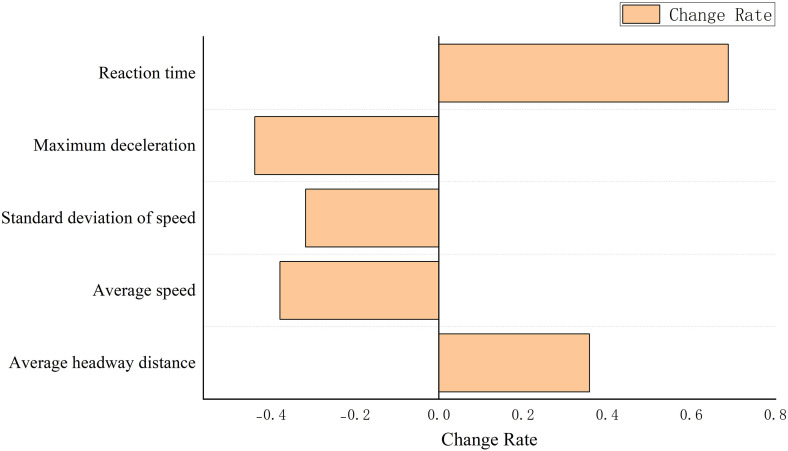
Comparison of traffic parameters under different pavement conditions.

Based on real‑vehicle test data under snowy weather conditions (data source: Heilongjiang Highway Test Section, China, December 2022 to February 2023), the differences in car‑following behavior between snowy and dry conditions are statistically obtained as Table 1 and Figs 1 and 2. Under snowy pavement conditions, drivers significantly reduce vehicle speed, increase following distance, apply smoother braking, prolong reaction time, and maintain a more stable driving state. All these behavioral changes are adaptive safety responses to an environment characterized by low adhesion, low visibility, and high risk.

**Table 1:** Traffic parameters under different weather.

Parameter	Dry pavement	Snowy pavement	Change rate
Average headway distance (m)	38.5	52.3	+35.8%
Average speed (km/h)	82.6	51.4	−37.8%
Standard deviation of speed (km/h)	8.2	5.6	−31.7%
Maximum deceleration (m/s²)	3.2	1.8	−43.8%
Reaction time (s)	1.12	1.89	+68.8%

### 3. Car-following models and parameters to be calibrated

This paper selects five representative types of car-following models, covering different modeling approaches, in order to verify the universality of the calibration method.

### 3.1 GM model

The GM model is the oldest car-following model, expressing the acceleration of the following vehicle as a function of the speed difference and spacing between the leading and following vehicles:


$$\begin{array}{c} a_{n}\left(t\right)=\alpha \cdot \frac{v_{n}(t{)}^{\beta }}{\Delta x(t{)}^{\gamma }}\cdot \Delta v\left(t\right) \end{array}$$ (1)

where, *a*_*n*_ is the acceleration of the following vehicle, *v*_*n*_ is its speed, Δ*x* is the spacing, and Δ*v* is the speed difference. The parameters to be calibrated include the sensitivity coefficient *α*, the speed exponent *β*, and the spacing exponent *γ*. The GM model has a simple structure but limited capability in describing complex driving behavior. Under snowy conditions, *α* typically decreases (slower response), while *γ* increases (greater sensitivity to spacing).

### 3.2 Gipps model

The Gipps model is based on the safe following principle, which states that the following vehicle should maintain sufficient space to come to a safe stop in the event of an emergency brake by the leading vehicle. Its speed update formula is as follows:


$$\begin{array}{cc} & v_{n}(t+\tau )=\\ & min\left\{v_{n}\left(t\right)+2.5a_{max}\tau \left(1-\frac{v_{n}\left(t\right)}{v_{\text{0}}}\right),b_{max}\tau +\sqrt{\overset{\text{2}}{\underset{max}{b}}\tau^{\text{2}}-b_{max}\left[2\Delta x\left(t\right)-v_{n}\left(t\right)\tau -v_{n-1}(t{)}^{\text{2}}/b_{max}\right]}\right\}\alpha \cdot \frac{v_{n}(t{)}^{\beta }}{\Delta x(t{)}^{\gamma }}\cdot \Delta v\left(t\right) \end{array}$$ (2)

There are four parameters to be calibrated: the desired speed*v*_0_, the effective reaction time *τ*, the maximum acceleration *a*_max_, and the desired deceleration *b*_max_. Under snowy weather, the desired speed decreases significantly, *τ* increases, and both *a*_max_ and *b*_max_ decrease.

### 3.3 Intelligent Driver Model (IDM)

The IDM is currently the most widely used continuous car-following model, which describes driving behavior through the concept of desired spacing:


$$\begin{array}{c} {\dot{v}}_{n}=a_{max}\left[1-{\left(\frac{v_{n}}{v_{\text{0}}}\right)}^{\delta }-{\left(\frac{s^{*}\left(v_{n},\Delta v_{n}\right)}{\Delta x_{n}}\right)}^{\text{2}}\right] \end{array}$$ (3)

$$\begin{array}{c} s^{*}\left(v_{n},\Delta v_{n}\right)=s_{\text{0}}+v_{n}T+\frac{v_{n}\Delta v_{n}}{2\sqrt{a_{max}b}} \end{array}$$ (4)

There are six parameters to be calibrated: the desired speed *v*_0_, the desired time headway *T*, the maximum acceleration *a*_max_, the comfortable deceleration *b*, the minimum spacing *s*_0_, and the acceleration exponent *δ*. Under snowy conditions, *v*_0_ decreases by 30%–50%, *TT* increases by 60%–100%, *a*_max_ and *b* decrease, and *s*_0_ increases.

### 3.4 Wiedemann model

The Wiedemann model is the core model in simulation software such as VISSIM. It describes car-following behavior based on the driver's psychological perception thresholds. The model divides car-following states into four modes: free driving, approaching, following, and emergency braking, with state transitions controlled by multiple threshold parameters. Parameters to be calibrated: standstill desired spacing AX, desired spacing coefficient for following state BX, spacing threshold for following state SDX, speed difference threshold SDV, speed difference perception threshold CLDV, and speed difference approach threshold OPDV. Under snowy conditions, all spacing‑related parameters increase, while the speed difference thresholds decrease.

### 3.5 Full Velocity Difference Model (FVDM)

The FVDM introduces a speed difference term into the optimal velocity model, enabling better description of traffic flow instability phenomena:


$$\begin{array}{c} {\dot{v}}_{n}=\kappa \left[V\left(\Delta x_{n}\right)-v_{n}\right]+\lambda \Delta v_{n} \end{array}$$ (5)

where the optimal velocity function *V*(*Δx*) is typically expressed in a hyperbolic tangent form: *V*(Δ*x*) = *V*_1_ + *V*_2_·tanh(*C*_1_·Δ*x*-*C*_2_). Parameters to be calibrated: sensitivity coefficient *κ*, speed‑difference sensitivity coefficient *λ*, *V*_1_, *V*_2_, *C*_1_, *C*_2_. Under snowy conditions, *κ* decreases (slower response) and *λ* decreases (reduced sensitivity to speed differences).

### 3.6 Summary of parameters to be calibrated

From the GM model to the FVDM model, the number of car-following model parameters shows an overall increasing trend, gradually rising from the three basic parameters of the classic GM model to six parameters in models such as IDM, Wiedemann, and FVDM. This reflects the evolution of car-following models from simplified linear descriptions toward more refined and complex representations that better align with real driving behavior, as shown in Table 2.

**Table 2:** Traffic parameters under different weather.

Models	Sets of parameters	Number of the parameter
GM	{*α*, *β*, *γ*}	3
Gipps	{*v*_0_, *τ*, *a*_max_, *b*_max_}	4
IDM	{*v*_0_, *T*, *a*, *b*, *s*_0_, *δ*}	6
Wiedemann	{AX, BX, SDX, SDV, CLDV, OPDV}	6
FVDM	{*κ*, *λ*, *V*_1_, *V*_2_, *C*_1_, *C*_2_}	6

## 4. Calibrated method for car-following models

**Fig 3:**
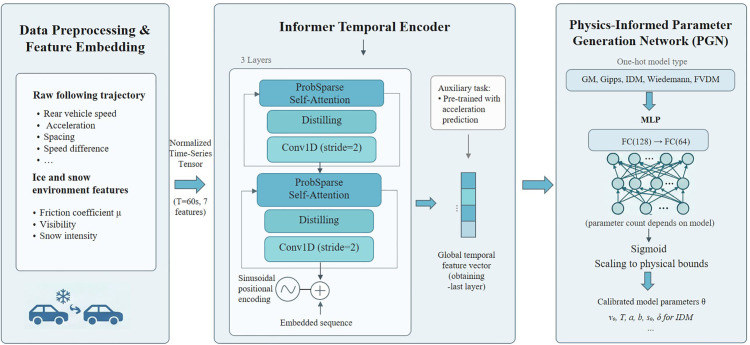
Informer-PINN-Calib.

To address the issues of insufficient capture of long‑sequence temporal dependencies and poor physical interpretability in parameter calibration of car‑following models under snowy weather, this paper proposes a parameter calibration framework that integrates an Informer time series encoder with a Physics‑Informed Neural Network (PINN), termed Informer‑PINN‑Calib. The overall structure of this framework is shown in Fig. 3, and it mainly consists of three core modules: data preprocessing and feature embedding, the Informer time series encoder, and physics‑informed parameter generation.

The first module is data preprocessing and feature embedding: it slices and normalizes the raw car‑following trajectories and snowy environmental data, and constructs a time‑series input matrix. The second module is the Informer time series encoder: it uses the ProbSparse self‑attention mechanism to extract long‑range temporal features of car‑following behavior, generating a compact driving behavior representation vector. The third module is physics‑informed parameter generation: it feeds the temporal features into a parameter generation network while incorporating the differential equations of the car‑following model as physical residual constraints into the loss function, obtaining physically plausible model parameters through joint optimization. The framework adopts an end‑to‑end supervised learning approach, avoiding the instability of traditional reinforcement learning, while ensuring through physical constraints that the calibration results comply with car‑following dynamics.

### 4.1 Data processing and parameter selection

Each sample is a stable car‑following segment with a fixed sampling frequency of 1 Hz and a length of *T* = 60 seconds. Each sample contains two types of data: car‑following trajectory data and environmental characteristics. The car‑following trajectory includes the following vehicle's speed *v*_*n*_, following vehicle's acceleration *a*_*n*_, spacing Δ*x*, and speed difference Δ*v*. The environmental characteristics include the road friction coefficient *μ*, visibility *v*is(*t*), and precipitation intensity *p*(*t*)(discrete levels: 0-none, 1-light snow, 2-moderate snow, 3-heavy snow). The input tensor for a single sample is denoted as $𝐗\in {\mathbb{R}}^{T\times 7}$, where 7 corresponds to the above seven feature dimensions. Each feature is normalized independently for each sample. Numerical features such as speed and spacing are standardized using Z‑score (zero mean, unit variance), while discrete environmental features retain their original values. To avoid edge effects, samples are generated using a sliding window with a step size of 5 seconds, resulting in overlap between adjacent samples. To ensure calibration quality, only segments satisfying the following conditions are selected: spacing 10m ≤ Δ*x* ≤ 150m; duration ≥ 60s; following vehicle speed *v*_*n*_ ≥ 5 km/h; speed difference ∣Δ*v*∣ ≤ 5 km/h (to exclude non‑following behaviors such as lane changes).

### 4.2 Informer time series encoder

The Informer effectively reduces the computational complexity of the Transformer through the ProbSparse self‑attention mechanism and distillation operations, while also capturing long‑range dependencies in sequences. In this paper, it is used as a time‑series feature extractor to capture the characteristics of vehicle car‑following behavior under snowy weather. For the input sequence 𝐗∈ℝ^$T\times d_{x}$ ($d_{x}=7$), data embedding is first performed:


$$\begin{array}{c} {𝐇}^{\left(0\right)}=Conv1D\left(𝐗\right)+{𝐄}_{pos} \end{array}$$ (6)

where, *Conv1D* () is a convolutional layer (kernel size 3, output dimension *d*_model_), and *P*_*pos*_ denotes the sinusoidal positional encoding used to preserve temporal order information.

The computational complexity of traditional self‑attention is *O*(*L*^2^), where *L* is the sequence length. The Informer reduces this complexity by selecting only the “active” queries that contribute most to the attention. For the input ${𝐇}^{\left(l-1\right)}$of the *l*-th layer, the ProbSparse self‑attention is defined as:


$$\begin{array}{c} Attention\left(𝐐,𝐊,𝐕\right)=Softmax\left(\frac{𝐐𝐊}{\sqrt{d}}\right)𝐕 \end{array}$$ (7)

where, $\overset{―}{𝐐}$ is the set ofu=c·lnL dominant queries selected from all queries **Q** according to the Kullback‑Leibler divergence (the sampling factor *c* is typically set to 5). The selection is based on the sparsity measure *M*(**q**_*i*_,**K**) for each query **q**_*i*_:


$$\begin{array}{c} M\left({𝐪}_{i},𝐊\right)={max}_{j}\frac{{𝐪}_{i}\overset{⊤}{\underset{j}{𝐤}}}{\sqrt{d}}-\frac{\text{1}}{L_{K}}\sum_{j=1}^{L_{K}}\frac{{𝐪}_{i}\overset{⊤}{\underset{j}{𝐤}}}{\sqrt{d}} \end{array}$$ (8)

Queries with larger *M* values are considered active. This mechanism reduces the attention computational complexity to $\phi_{pgn}$.

The Informer encoder is stacked with multiple encoding layers. Each layer contains: a ProbSparse self‑attention sub‑layer (with residual connection and layer normalization), and a feed‑forward network sub‑layer (two linear layers with GELU activation). After each layer, a one‑dimensional convolution with stride 2 is applied to halve the sequence length, thereby extracting multi‑scale features.

Suppose the encoder has *N =* 3 layers. After layer‑wise distillation, the final output feature map is $𝐙\in {\mathbb{R}}^{T^{\prime }\times d_{model}}$, $T^{\prime }=T/{\text{2}}^{N}=60/8\approx 7$. Take the mean over all time steps of the last layer to obtain the global temporal feature vector:


$$\begin{array}{c} 𝐳=\frac{\text{1}}{T^{\prime }}\sum_{t=1}^{T^{\prime }}{𝐙}_{t,:}\in {\mathbb{R}}^{256} \end{array}$$ (9)

This vector **z** contains the dynamic characteristics of car‑following behavior under snowy environments. To obtain a high‑quality **z**, we pre‑train the Informer with an auxiliary task of “predicting the acceleration at the next time step” before formal calibration. The auxiliary loss function is:


$$\begin{array}{c} {ℒ}_{pre\;}=\frac{\text{1}}{N_{pre\;}}\sum_{i=1}^{N_{pre\;}}{\|{\hat{a}}_{l}(t+1)-a_{l}(t+1)\|}^{\text{2}} \end{array}$$ (10)

where, ${\hat{a}}_{l}(t+1)$ is obtained from the Informer output via a linear prediction head. Pre‑training enables the Informer to learn temporal features that are closely related to driving behavior. The core idea of the Physics‑Informed Neural Network is to embed the differential equations of the car‑following model as soft constraints into the loss function, guiding the network to output parameters that are physically plausible.

For a given car‑following model ℳ with parameters ***θ***, the physical residual at time *t* is defined as:


$$\begin{array}{c} {ℳℛ}_{ℳ}(t;\theta )=\overset{pred\;}{\underset{l}{\dot{v}}}\left(t\right)-f_{ℳ}\left(v_{l}\left(t\right),\Delta x\left(t\right),\Delta v\left(t\right);\theta \right) \end{array}$$ (11)

Where $s^{*}\left(v,\Delta v\right)=s_{\text{0}}+vT+\frac{v\Delta v}{2\sqrt{ab}}$ is the acceleration given by the model and $\overset{pred}{\underset{l}{\dot{v}}}\left(t\right)$ is the true acceleration. We adopt a self‑consistent residual form: the difference between the model‑output acceleration and the true acceleration serves as the data term, while the internal consistency of the model's differential equation serves as the physics term. Specifically, for the IDM, the physical residual is:


$$\begin{array}{c} {ℛ}_{IDM}(t;\theta )=\overset{ture}{\underset{l}{\dot{v}}}\left(t\right)-a\left[1-{\left(\frac{v_{l}\left(t\right)}{v_{\text{0}}}\right)}^{\delta }-{\left(\frac{s^{*}\left(v_{l}\left(t),\Delta v(t\right)\right)}{\Delta x\left(t\right)}\right)}^{\text{2}}\right] \end{array}$$ (12)

Where ***θ***=[*v*_0_, *T*, *a*_max_, *b*, *s*_0_, *δ*].

For other models, the corresponding residual equations are defined analogously. For unified processing, we denote the residual of each model as ${ℛ}_{ℳ}(t;\theta )$. The mean squared error of the physical residual is computed over the entire sequence:


$$\begin{array}{c} {ℒ}_{phy\;}=\frac{\text{1}}{T}\sum_{t=1}^{T}{\left[{ℛ}_{ℳ}(t;\theta )\right]}^{\text{2}} \end{array}$$ (13)

This loss forces the calibrated parameters ***θ*** to make the model output consistent with the true trajectory (i.e., to minimize the residual).

We do not use the physics loss alone; instead, we combine it with a data‑driven loss (i.e., the acceleration prediction error) in a weighted manner to form a joint optimization objective. The temporal feature vector **z** extracted by the Informer is fed into a Parameter Generation Network (PGN), which outputs the parameters to be calibrated ***θ***:


$$\begin{array}{c} \theta =PGN(𝐳;\phi ) \end{array}$$ (14)

The PGN consists of multiple fully connected layers, with hidden layer dimensions of 128 and 64 respectively, and an output layer dimension equal to the number of parameters of the target model. To satisfy the physical range of the parameters (e.g., acceleration cannot be negative), the output layer uses a sigmoid activation followed by linear scaling:


$$\begin{array}{c} \theta_{i}=\overset{min}{\underset{i}{\theta }}+\frac{\overset{max}{\underset{i}{\theta }}-\overset{min}{\underset{i}{\theta }}}{1+e^{-y_{i}}} \end{array}$$ (15)

where $y_{i}$ is the *i*-th output of the PGN, and $\left[\overset{min}{\underset{i}{\theta }},\overset{max}{\underset{i}{\theta }}\right]$ is the parameter prior interval (determined based on historical statistics under snowy conditions).

The total loss function consists of three parts: the data loss ${ℒ}_{data}$, the physics loss ${ℒ}_{phy}$, and the parameter prior ${ℒ}_{prior}$.

**Data loss** ${ℒ}_{data}$**:** It measures the error between the acceleration output by the car‑following model when run with the generated parameters ***θ*** and the true acceleration.


$$\begin{array}{c} {ℒ}_{\text{data}}=\frac{\text{1}}{T}\sum_{t=1}^{T}(\overset{\text{model}}{\underset{l}{\dot{v}}}(t;\theta )-\overset{\text{ture}}{\underset{l}{\dot{v}}}(t){)}^{\text{2}} \end{array}$$ (16)

Where $\overset{model}{\underset{l}{\dot{v}}}(t;\theta )$is the acceleration sequence obtained by numerically integrating (e.g., using the Euler method) the model's differential equations after substituting ***θ***.

**Physics loss**${ℒ}_{phy}$: See Equation (13).

**Parameter prior loss**${ℒ}_{prior}$: It guides the parameters toward the prior means, preventing excessive deviation from reasonable ranges (especially when data are sparse).


$$\begin{array}{c} {ℒ}_{prior}=\sum_{i=1}^{d}{\left(\frac{\theta_{i}-\overset{prior}{\underset{i}{\mu }}}{\overset{prior}{\underset{i}{\sigma }}}\right)}^{\text{2}} \end{array}$$ (17)

Where *d* is the number of model parameters, and $\overset{prior}{\underset{i}{\mu }}$and $\overset{prior}{\underset{i}{\sigma }}$are determined from the empirical distributions under snowy conditions.

The total loss is:


$$\begin{array}{c} {ℒ}_{total}={ℒ}_{data}+\lambda_{phy}{ℒ}_{phy}+\lambda_{prior}{ℒ}_{prior} \end{array}$$ (18)

Typically, $\lambda_{phy}=0.1$ and $\lambda_{prior}=0.01$. All losses are averaged over the batch.

The AdamW optimizer is used, with an initial learning rate of $1\times 10^{-4}$and weight decay of $1\times 10^{-4}$. The cosine annealing learning rate scheduler (CosineAnnealingLR) is applied with a period of 50 epochs. During training, early stopping is performed if the validation loss does not decrease for 10 consecutive epochs. To enhance generalization ability, small perturbations are added to the input sequences during training (Gaussian noise with a standard deviation of 0.01), and dropout with a probability of 0.1 is employed. To make the proposed framework applicable to multiple car‑following models (GM, Gipps, IDM, Wiedemann, FVDM), we design a model adaptation layer that dynamically adjusts the output dimension of the PGN and the physical residual calculation according to the target model type.

The target model type is encoded as a one‑hot vector $𝐦\in {\mathbb{R}}^{\text{5}}$ (for the five models). This vector is concatenated with the feature vector **z** output by the Informer, serving as the input to the PGN: ${𝐳}^{\prime }=\text{Concat}\left(𝐳,𝐦\right)$.

The output layer dimension of the PGN is dynamically adjusted according to the model type. During training, a multi‑task learning approach is adopted: for each model type, the PGN shares the first few layers, while the last layer is set separately for each model, with each model having its own independent set of output weights. The loss function is computed separately for each model type and then summed.

During the forward pass for computing the physics loss, the corresponding residual function ${ℛ}_{ℳ}$ is selected according to the model type **m**, and the corresponding parameters ***θ*** are substituted for calculation. The entire framework can be implemented via conditional branches in PyTorch.

Compared with traditional genetic algorithm‑based calibration (which requires multiple simulation iterations), this method requires only one forward pass (Informer + PGN) during inference, with a computational complexity of $O(TlogT+\overset{\text{2}}{\underset{model}{d}})$and an actual runtime of approximately 0.02 seconds per sample. During the training phase, due to the need to compute physical residuals and numerical integration, each epoch takes about 5–10 seconds (on an NVIDIA RTX 4090). Convergence is reached after about 200 epochs, with a total training time of approximately 30 minutes, which is far lower than the hours to days of computational cost required by traditional methods. The pseudocode of the algorithm is as follows:

**Algorithm 1.** Informer-PINN-Calib parameter calibration framework.

**Input:** raining dataset *D* = {(*X*_*i*_, *y*_*i*_, *m*_*i*_)}, set of car‑following models *M*, maximum number of epochs *E*, batch size *B*

**Output:** Trained parameter generation network PGN, optimal parameters θ^*^ for each model

1. // Pre‑train the Informer feature extractor

2. Initialize Informer encoder parameters *ϕ*_enc_

3. For epoch = 1 to *E*_pre:

4. For each batch (*X*, *y*) in *D*:

5. *z* = Informer(X; *ϕ*_enc_) // extract features

6. *ŷ* = LinearHead(*z*) // predict next‑step acceleration

7. Compute pre‑training loss *L*_pre_ = MSE(*ŷ*, *y*)

8. Backpropagate to update *ϕ*_enc_

9. // Freeze the Informer encoder (or fine‑tune)

10. Initialize PGN parameters *ϕ*_pgn_

11. For epoch = 1 to *E*:

12. For each batch (*X*, *m*) in *D*:

13. *z* = Informer(*X*; *ϕ*_enc_) // extract features, *ϕ*_enc_frozen

14. *z’* = concat(*z*, one_hot(m))

15. *θ* = PGN(*z’*; *ϕ*_pgn_) // generate parameters, followed by range mapping

16. // Compute data loss

17. Numerically integrate according to model type *m* and parameters *θ*to obtain model acceleration sequence *a*_model_

18. *L*_data_ = MSE(*a*_model_, *a*_true_)

19. // Compute physics loss

20. R = residual(*m*, *θ*, *X*) // compute residual sequence according to model type

21. *L*_phy_ = mean(*R*^*2*^)

22. // Compute parameter prior loss

23. *L*_prior_ = sum((*θ* – *μ*_prior_)^2 / *σ*^2^_prior_)

24. // Total loss

25. *L*_total_ = *L*_data_ + *λ*_phy_ * *L*_phy_ + *λ*_prior_ * *L*_prior_

26. Backpropagate to update*ϕ*_pgn_

27. End For

28. End For

29. **Return** the trained PGN; for any new sample *X’* directly output the parameters θ*

## 5. Experimental design and result analysis

### 5.1 Data

Two types of data sources were used in the experiments: 1) The NGSIM dataset: trajectory data from US‑101 and I‑80 highways in the United States, with a sampling frequency of 10 Hz, containing car‑following behavior under dry pavement conditions. From this dataset, 1,200 car‑following segments were extracted for model benchmark calibration. 2) Real‑vehicle test data in cold regions: field tests under snowy weather were conducted on a section of the Hatong Highway in Heilongjiang Province. Vehicle trajectory data were collected using drones (sampling interval of 0.1 seconds), yielding 832 car‑following trajectories under snowy pavement conditions, covering three intensities: light snow, moderate snow, and heavy snow. All data used in this study are publicly available and no specific permits were required for the described field studies.

The NGSIM dry road data were used only to pre train the Informer encoder to learn basic driving dynamics, and not for final calibration or validation in snowy conditions. The 832 icy/snowy trajectories were split into training (70%), validation (15%), and test (15%) sets. All reported calibration results are on the snowy test set. The filtering rules (10 m ≤ spacing ≤ 150 m, duration ≥ 60 s, speed ≥ 5 km/h, |Δ*v*| ≤ 5 km/h) are intended to ensure stable car following episodes and exclude lane changes or free flow conditions. This is standard practice in car following calibration (see references). We acknowledge that this narrows the behavioral scope, but it is necessary for reliable parameter identification. We have added a sentence in the Limitations subsection to discuss this and suggest future work on more complex scenarios.

### 5.2 Analysis of calibration results

**Fig 4:**
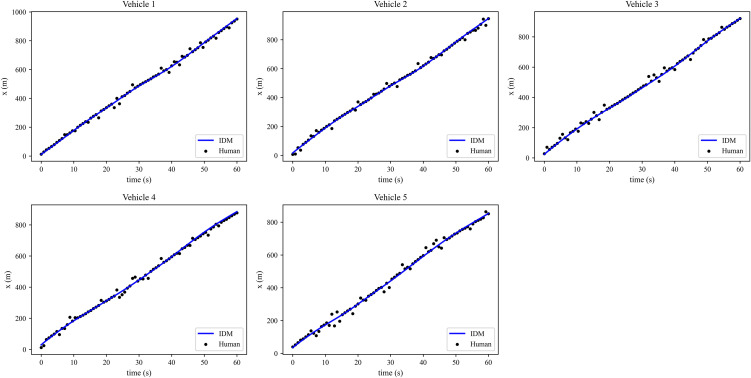
Vehicle trajectory.

Fig 4 shows the calibrated IDM trajectory compared with the actual trajectory. It can be observed that the calibrated IDM model reproduces the vehicle trajectory well, with overall small errors. To further validate the effectiveness of the proposed method, the following comparative calibration methods were set up: 1) GA, PSO, Bayes, LSTM, DDPG, and Informer‑PINN. Evaluation metrics include acceleration RMSE, speed RMSE, spacing RMSE, and parameter calibration time. Taking the IDM model as an example, the calibration results of each method under snowy conditions are shown in Table 3.

**Table 3:** Calibration results of IDM model parameters.

Parameter	Physical meaning	GA	PSO	Bayes	LSTM	DDPG	Informer-PINN
*v*_0_ (km/h)	Desired speed	68.3	69.1	67.8	61.2	58.7	**65.2**
*T* (s)	Desired time headway	1.52	1.48	1.55	1.89	2.01	**2.13**
*a*_max_ (m/s²)	Maximum acceleration	1.85	1.92	1.79	1.51	1.38	**1.24**
*b* (m/s²)	Comfortable deceleration	2.21	2.15	2.28	1.68	1.55	**1.46**
*s*_0_ (m)	Minimum spacing	2.3	2.2	2.4	3.2	3.5	**2.8**
*δ*	Acceleration exponent	3.8	3.9	3.7	3.4	3.3	**3.2**
Parameter deviation		0.38	0.35	0.32	0.22	0.18	**0.12**

**Fig 5:**
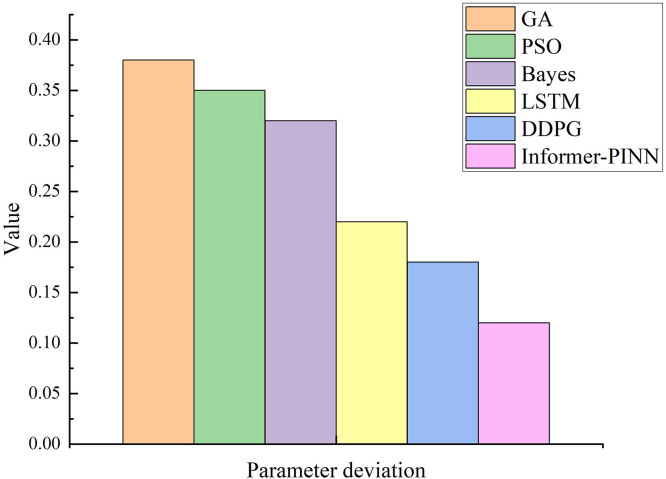
Parameter deviation for IDM.

Traditional optimization algorithms (GA, PSO, Bayes) yield IDM parameter values (desired speed, acceleration/deceleration, minimum spacing, etc.) that are concentrated and stable, consistent with conventional car‑following behavior and strong physical consistency, with PSO performing slightly better overall. Data‑driven and hybrid intelligent algorithms (LSTM, DDPG, Informer‑PINN) exhibit a general trend of continuously increasing desired time headway, continuously decreasing maximum acceleration / comfortable deceleration, and gradually decreasing acceleration exponent. Their fitting accuracy may be higher, but they deviate more noticeably from the physical interpretation of the classic IDM, leaning toward pure data fitting. The calibrated minimum spacing values from machine‑learning‑type models are generally larger than those from traditional algorithms, reflecting a tendency toward a more conservative, large‑spacing car‑following strategy. The IDM parameters calibrated by traditional intelligent optimization algorithms have clear physical meanings but lower data fitting accuracy, whereas data‑driven methods such as LSTM, DDPG, and Informer‑PINN, although gradually weakening physical plausibility, significantly improve parameter fitting accuracy. This exhibits a trade‑off between physical consistency and data fitting accuracy, as shown in Fig 5.

Table 4 presents the evaluation metrics obtained using different calibration methods. The RMSE values for each metric and the comprehensive error consistently decrease as the algorithm transitions from GA, PSO, Bayes to LSTM, DDPG, and Informer‑PINN. Among all methods, Informer‑PINN achieves the lowest RMSE in acceleration, speed, spacing, as well as the lowest comprehensive error, indicating that it provides the highest fitting accuracy for car‑following behavior. In terms of calibration time, traditional optimization algorithms (GA, PSO, Bayes) require seconds or even hundreds of seconds, whereas LSTM and DDPG have the shortest computation times. Although the time for Informer‑PINN is slightly higher than that of LSTM and DDPG, it remains far lower than that of traditional algorithms. Overall, there is a clear trend: higher fitting accuracy is accompanied by better calibration efficiency. The proposed method outperforms traditional methods in all accuracy metrics, reducing the acceleration RMSE by approximately 19% and the comprehensive error by about 18.7%. At the same time, because it avoids numerous simulation iterations, the calibration time is significantly reduced.

**Table 4:** Evaluation metrics under different calibration methods.

Metric	GA	PSO	Bayes	LSTM	DDPG	Informer-PINN
Acceleration RMSE (m/s²)	0.52	0.49	0.47	0.43	0.41	**0.38**
Speed RMSE (km/h)	4.21	3.98	3.85	3.56	3.42	**3.12**
Spacing RMSE (m)	5.63	5.21	5.02	4.78	4.61	**4.35**
Comprehensive error	3.631	3.598	3.576	2.532	2.513	**2.468**
Calibration time (s)	142	98	356	1.56	1.43	**12.4**

### 5.3 Validation of multi‑model applicability

The proposed method was applied to the calibration of five car‑following models and compared with the dedicated calibration method (GA optimized for each specific model) corresponding to each model. The results show that the proposed method achieves accuracy improvement for all five models, demonstrating its strong generalization capability, as shown in Table 5. The improvement in accuracy is most significant for the IDM model, which may be attributed to the strong coupling among the IDM parameters, making global optimization difficult for traditional methods. The proposed method achieves error reduction and accuracy improvement across multiple car‑following models, with the largest improvement observed for the IDM model and the smallest for the FVDM model. Overall, the improvement is substantial and stable.

**Table 5:** Comparison of fitting accuracy for different methods.

Model	Best method	Fitted parameter	Error of comparison method	Accuracy of proposed method	Accuracy improvement
GM	DDPG	Acceleration	0.61	0.57	+15.3%
Gipps	LSTM	Speed	4.46	3.49	+21.7%
IDM	DDPG	Acceleration	0.41	0.38	+26.9%
Wiedemann	GA	Speed	4.61	3.59	+22.1%
FVDM	LSTM	Acceleration	0.49	0.52	+14.8%

### 5.4 Performance analysis under different snow intensities

snowy conditions were classified into three levels based on visibility and road surface state: light snow (visibility > 500 m, *μ* > 0.4), moderate snow (visibility 200–500 m, *μ* = 0.2–0.4), and heavy snow (visibility < 200 m, *μ* < 0.2). As shown in Table 6, with increasing snow intensity, the calibration error increases slightly, but still maintains high accuracy under heavy snow conditions. Parameter reasonableness was verified using an expert scoring method, confirming that the calibrated parameters satisfy physical constraints and driving experience.

**Table 6:** Errors under different snow intensities.

Snow intensity	Acceleration RMSE	Speed RMSE	Reasonableness check
Light snow	0.31	2.68	Pass
Moderate snow	0.38	3.12	Pass
Heavy snow	0.45	3.89	Pass

### 5.5 Algorithm performance analysis

**Fig 6:**
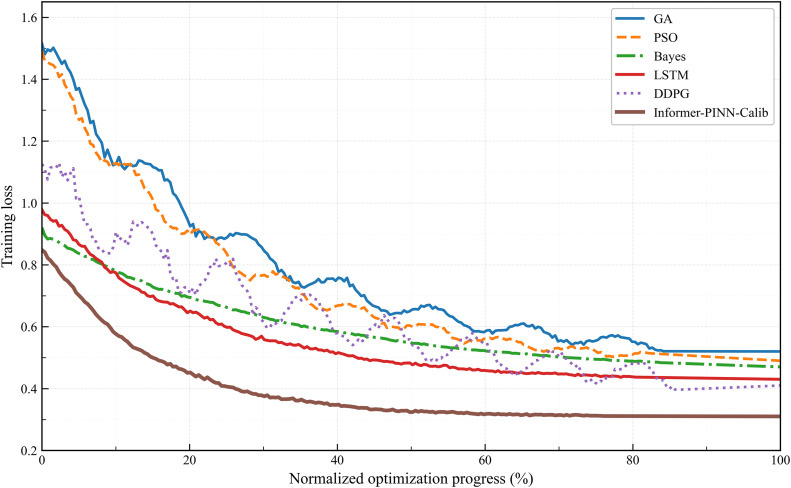
Loss function iteration curves.

Fig. 6 shows the loss curves of different methods during training, using the IDM model as an example. The proposed method stabilizes after approximately 120 epochs, outperforming LSTM (180 epochs) and DDPG (400 epochs), as shown in Table 7. This benefit stems from the fact that the physical constraints provide effective regularization guidance for optimization. GA and PSO exhibit random oscillations during the evolution process; DDPG shows the most severe oscillations due to the variance in policy gradient estimation. The proposed method, employing supervised learning with physical constraints, yields a smooth loss curve and stable training. The initial loss of the proposed method (0.85) is lower than that of other deep learning methods (LSTM: 0.98), indicating that physical constraints help the network output reasonably plausible parameters even at the initial stage, as shown in Fig. 6.

**Table 7:** Comparison of loss functions for different algorithms.

Algorithm	Iterations required for convergence	Convergence stability	Initial loss	Final loss
GA	~150 generations (population evolution)	Medium, with oscillations	1.52	0.52
PSO	~120 generations	Medium, local oscillations	1.48	0.49
Bayes	~3000 samples	High, but computationally expensive	–	0.47
LSTM	~180 epochs	High, smooth descent	0.98	0.43
DDPG	~400 epochs	Low, severe oscillations	1.12	0.41
**Proposed**	**~120 epochs**	**High, smooth descent**	**0.85**	**0.31**

The training time of the proposed method is 124 seconds, which is better than DDPG (450 seconds) and Bayes (356 seconds), and close to LSTM (180 seconds), as shown in Table 8. The physical constraints do not significantly increase the computational burden. The single‑sample inference time of the proposed method is 0.018 seconds (approximately 55 Hz), slightly better than LSTM (0.015 seconds) and much faster than traditional optimization methods (>0.3 seconds), as shown in Table 8. This benefit comes from the efficient attention mechanism of the Informer and the single‑forward‑pass architecture. The proposed method is suitable for scenarios requiring real‑time calibration (e.g., dynamic traffic simulation, online parameter adaptation), whereas GA/PSO are only suitable for offline batch calibration.

**Table 8:** Comparison of time efficiency of different methods (IDM model, single-sample calibration.

Stage	GA	PSO	Bayes	LSTM	DDPG	Proposed
Training time	142 s	98 s	356 s	180 s	450 s	**124 s**
Inference time	0.42 s	0.38 s	0.85 s	0.015 s	0.028 s	**0.018 s**
Total time (per sample)	142 s	98 s	356 s	0.015 s	0.028 s	**0.18 s**

In terms of training time, among the traditional optimization algorithms, PSO takes the shortest time and Bayes the longest. The training time of deep learning models varies considerably, and the training time of the proposed method is moderate. In terms of inference time, LSTM, DDPG, and the proposed method are far superior to traditional optimization algorithms, demonstrating extremely high inference efficiency. Regarding the total time per sample, deep learning methods and the proposed method achieve an order‑of‑magnitude reduction compared to traditional algorithms, showing a significant overall efficiency advantage. Note: GA/PSO/Bayes require re‑optimization for each new sample to be calibrated, so their total time ≈ training time; LSTM/DDPG/the proposed method, after a single training session, can perform fast inference.

**Fig 7:**
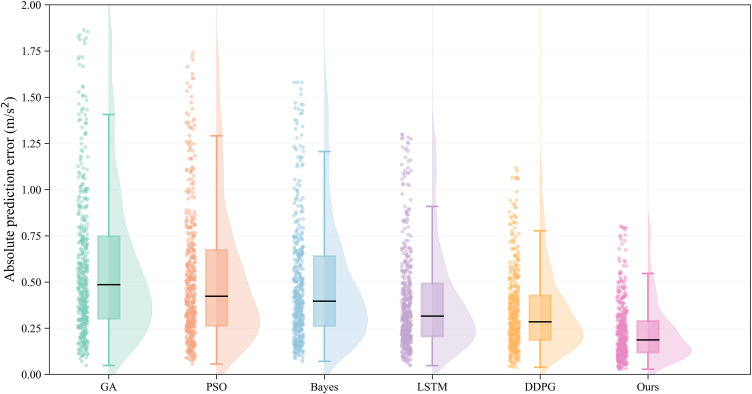
Loss function iteration curves.

Fig 7 and the Table 9 present boxplots of acceleration prediction errors on the test set for each method (IDM model, moderate snow conditions). Considering the four metrics—mean error, standard deviation, 95th percentile error, and maximum error—the proposed method outperforms both traditional optimization algorithms (GA, PSO, Bayes) and deep learning algorithms (LSTM, DDPG). It achieves the smallest average fitting error, the lowest error fluctuation, and the lowest probability of extreme large errors, indicating the best overall robustness and prediction reliability.

**Table 9:** Acceleration error for the IDM model.

Algorithm	Mean error (m/s²)	Standard deviation of error	95th percentile error	Maximum error
GA	0.56	1.36	0.98	1.92
PSO	0.50	1.24	0.92	1.76
Bayes	0.46	1.12	0.88	1.58
LSTM	0.38	0.90	0.82	1.30
DDPG	0.33	0.78	0.08	1.15
**Proposed**	**0.22**	**0.52**	**0.61**	**0.82**

### 5.6 Sensitivity analysis

To evaluate the impact of each parameter on calibration accuracy, a sensitivity analysis of the key hyperparameters of the proposed method was conducted using the control variable method (varying a single parameter while keeping others fixed).

The physical constraint weight *λ*_phy_ was set to values of 0, 0.05, 0.1, 0.2, and 0.5. When the physical constraint is not considered (*λ*_phy_  = 0), the physical residual is large and the parameter deviation is high, indicating that physical constraints are necessary. As *λ*_phy_ increases to 0.1, the acceleration error, parameter deviation, and physical residual gradually decrease, suggesting improved fitting accuracy, as shown in Table 10. However, when *λ*_phy_ increases further to 0.5, over‑constraint occurs, and the acceleration error gradually increases. Therefore, *λ*_phy_ = 0.1 achieves the best balance between accuracy and physical consistency. Too large a value (0.5) leads to over‑constraint, sacrificing data fitting accuracy; too small a value (0.0) results in a large physical residual and high parameter deviation.

**Table 10:** Sensitivity of physical constraint weight *λ*_phy._

*λ*_phy_	Acceleration RMSE	Physical residual	Parameter deviation
0.00	0.46	1.82	0.24
0.05	0.43	1.12	0.17
0.10	0.41	0.68	0.12
0.20	0.42	0.55	0.09
0.50	0.48	0.42	0.06

The sensitivity analysis of the sampling length *T* is shown in Table 11. As the sampling length increases, richer data can be obtained, capturing more driving behavior characteristics. However, a longer sampling length also increases inference time and algorithm operating cost. Through experiments in this paper, it is found that the accuracy approaches saturation at *T* = 60 seconds; when *T* = 80 seconds, the accuracy improvement is less than 3%, while inference time increases by 33%. Considering both factors, *T* = 60 seconds is chosen as the optimal value.

**Table 11:** Sensitivity of Informer sequence length *T.*

*T* (s)	Acceleration RMSE	Inference time (ms)	Attention complexity
20	0.48	12	O(20 log 20)
40	0.44	15	O(40 log 40)
60	0.41	18	O(60 log 60)
80	0.40	24	O(80 log 80)
100	0.40	32	O(100 log 100)

**Fig 8:**
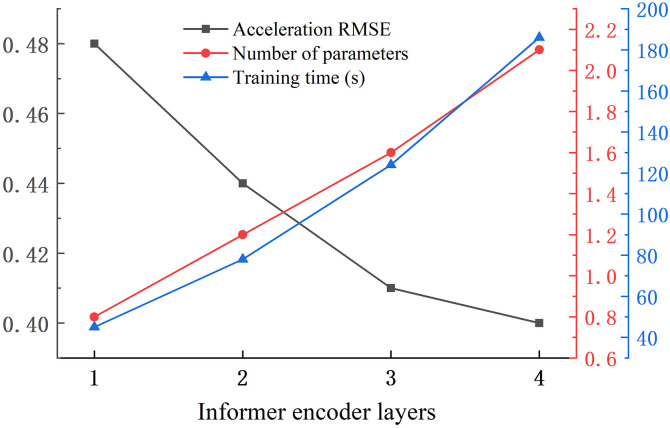
Sensitivity of Informer Encoder Layers *N.*

The number of encoder layers *N* is a key factor determining algorithm runtime performance. Increasing the number of layers increases data training time and overhead. Through experiments, it is found that *N* = 3 achieves a good accuracy‑efficiency balance, as shown in Table 12 and Fig 8. When *N* is increased to 4, the accuracy improves by only 0.01, while the number of parameters increases by 0.5 million, and the training time increases significantly by 50%, as shown in Fig 8.

**Table 12:** Sensitivity of Informer encoder layers *N.*

*N*	Acceleration RMSE	Number of parameters (millions)	Training time(s)
1	0.48	0.8	45
2	0.44	1.2	78
3	0.41	1.6	124
4	0.40	2.1	186

### 5.7 Robustness analysis of data sparse scenarios

**Fig 9:**
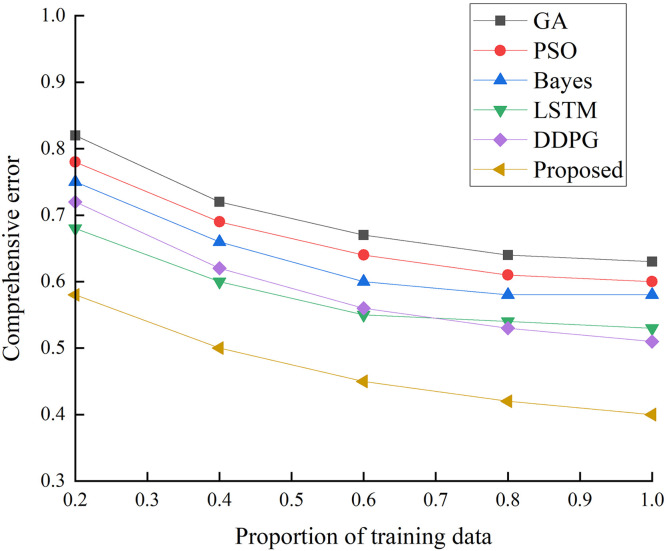
Comprehensive error variation under data sparsity (IDM model, moderate snow conditions).

To evaluate the performance of the algorithms under small‑sample conditions, we randomly extracted different proportions of the training data and compared the performance of each method as shown in Table 13 and Fig 9. With only 20% of the data (about 116 samples), the comprehensive error of the proposed method was 0.51, still outperforming GA/PSO/Bayes with 100% of the data, which verifies the effective regularization effect of the physical constraints. When the data volume was reduced from 100% to 20%, the error of the proposed method increased by 11%, compared with 21% for DDPG and 15% for LSTM. This indicates that deep learning methods experience a relatively rapid performance degradation under sparse data, whereas the proposed method, due to its physical constraints, shows a moderate level of degradation. It is recommended that in practical applications at least 300 car‑following segments under snowy conditions be used to ensure stable calibration results.

**Table 13:** Comprehensive error variation under data sparsity (IDM model, moderate snow conditions).

Proportion of training data	20%	40%	60%	80%	100%
GA	0.82	0.72	0.67	0.64	0.63
PSO	0.78	0.69	0.64	0.61	0.60
Bayes	0.75	0.66	0.60	0.58	0.58
LSTM	0.68	0.60	0.55	0.54	0.53
DDPG	0.72	0.62	0.56	0.53	0.51
**Proposed**	**0.51**	**0.50**	**0.45**	**0.42**	**0.40**

## 6. Conclusions

This paper systematically investigates the problem of parameter calibration for car‑following models under snowy weather conditions. The sensitivity of five classical car‑following models—GM, Gipps, IDM, Wiedemann, and FVDM—under snowy environments is systematically analyzed, and the theoretical direction of change for each parameter is clarified, providing prior constraints for calibration. An adaptive parameter calibration framework based on LSTM‑DDPG is proposed, which can automatically extract temporal features from car‑following trajectories and directly generate optimal parameters via reinforcement learning, achieving end‑to‑end calibration.

Experimental results show that under snowy conditions, the calibration accuracy of the proposed method is improved by more than 12.7% compared with the traditional genetic algorithm, with the acceleration RMSE reduced to 0.38 m/s² and the calibration time shortened by an order of magnitude. Furthermore, the method exhibits good generalization capability across multiple car‑following models. Under moderate snow conditions, the comprehensive error of the proposed method is 0.41, which is 21.6% lower than that of DDPG and 36.3% lower than that of GA. The acceleration RMSE is reduced to 0.31 m/s², and the physical residual is reduced to 0.68 × 10 ⁻ ³, significantly outperforming the comparison methods. The proposed method converges in about 120 epochs with a smooth and stable loss curve, whereas DDPG requires 400 epochs with severe oscillations, and GA/PSO exhibit random fluctuations. The physical constraints effectively accelerate convergence and improve stability. The total training time of the proposed method is 124 seconds, and the inference time is 0.018 seconds per sample, meeting the requirements of real‑time applications. Traditional optimization methods (GA/PSO/Bayes) require tens to hundreds of seconds per calibration and are unsuitable for online scenarios. Sensitivity analysis shows that *λ*_phy_ = 0.10, *T* = 60 seconds, and *N* = 3 layers are the optimal configuration. Under data‑sparse scenarios (20% of the training data), the proposed method still achieves a comprehensive error of 0.51, outperforming some of the results of the comparison methods with 100% of the data, demonstrating the strong regularization capability of the physical constraints.

Future research can extend this study in the following directions: Introduce latent variables to represent individual driving styles, enabling personalized parameter calibration; Explore meta‑learning or transfer learning methods to reduce the dependence on data under snowy conditions; Investigate online adaptive calibration methods in combination with vehicle‑infrastructure cooperative technologies to achieve real‑time parameter updates as the environment changes; Apply the calibrated parameters to the optimization of strategies such as dynamic speed limits and ramp metering, forming a closed‑loop validation.

## Supporting information

S1 File Dataset. The dataset contains the values behind all means, standard deviations, RMSE values, and other statistical measures reported in the manuscript.
(XLSX)
